# Ecological Insights From Camera Trapping Span Biological Taxa, and the Globe

**DOI:** 10.1002/ece3.70947

**Published:** 2025-02-02

**Authors:** Jason T. Fisher

**Affiliations:** ^1^ School of Environmental Studies University of Victoria Victoria British Columbia Canada

## Abstract

Camera trap research has grown to encompass the globe, with applications in terrestrial, marine, and aquatic environments. Insights on plant, invertebrate, and vertebrate communities are rapidly expanding our knowledge of ecological systems.

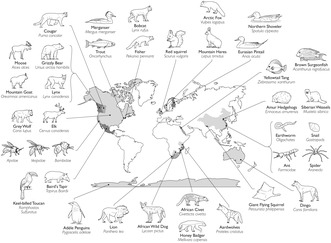

A technological revolution in ecological research was launched by advancements in camera trapping (Kucera and Barrett 2011; O'Connell, Nichols, and Karanth 2011). “Trapping” an observation of a species in its environment at a fixed place and time has allowed scientists to sample a widening range of taxa and ecosystems. With the application of appropriate sampling designs and statistical models (Burton et al. 2015), scientists have been able to answer questions never before possible. Mammal ecologists were early adopters, taking advantage of passive heat‐in‐motion detectors developed in the early 2000s to sample homeotherms (Finn 2005; Vercauteren, Smith, and Stevenson 2005). The development of larger memory cards and long‐lasting power enabled the use of repeated time‐lapse photography for ectotherms and vegetation, and now the ecological insights derived from camera traps span biological taxa and ecological hierarchies. In this special issue of *Ecology and Evolution*, we invited camera‐trap papers from around the globe to showcase the depth and breadth of camera‐trap applications. We particularly aimed for representation from the global south, which has rich and heretofore relatively untapped potential compared to the global north (Steenweg et al. 2017; Agha et al. 2018; Fisher 2023; Mugerwa et al. 2024). The papers in this special issue showcase some of the great versatility of camera‐trap research and the scientific advancements they offer (Figure 1).

**FIGURE 1:**
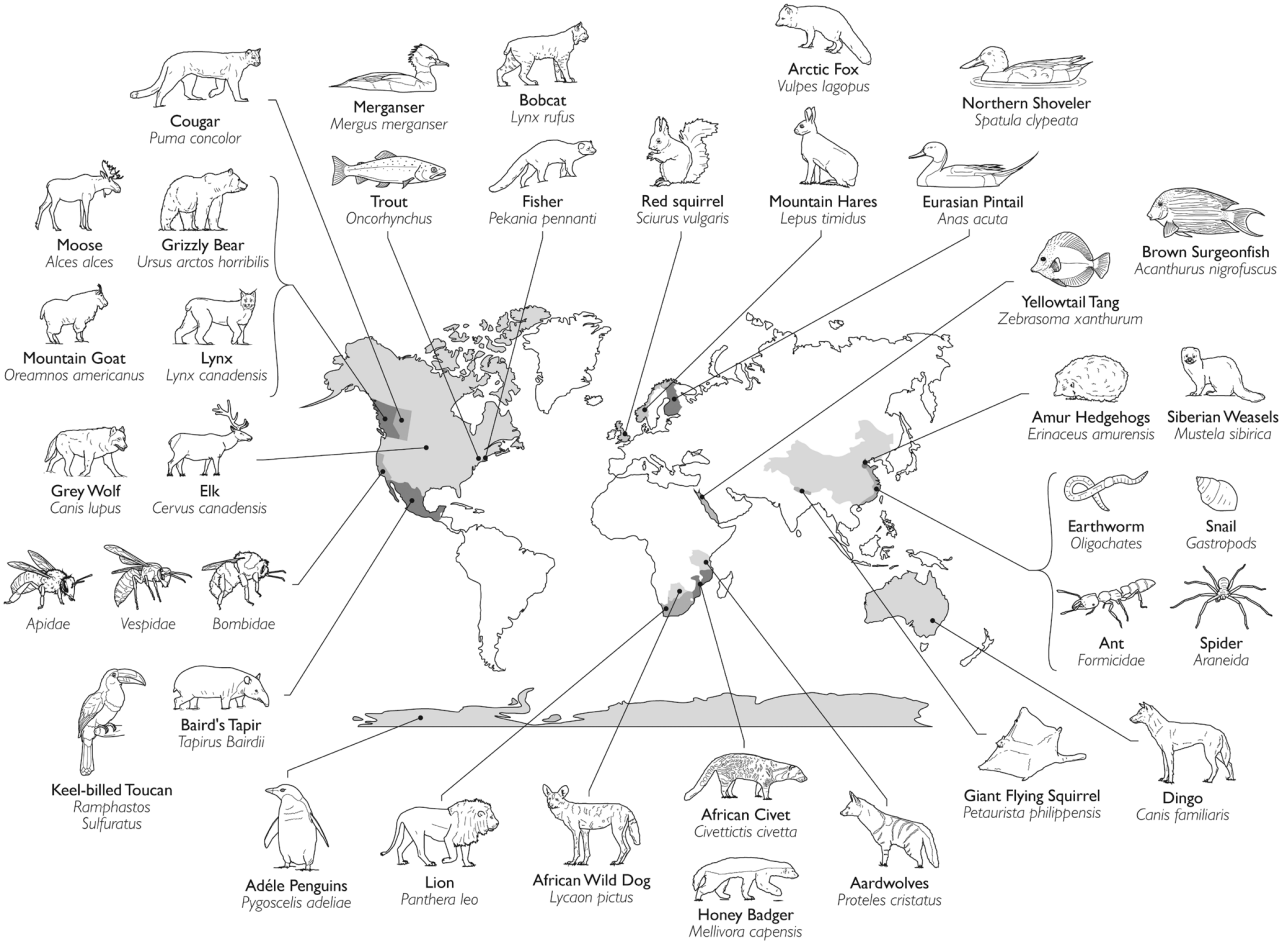
Research articles in this Special Issue “Ecological Insights in Camera Trapping” in *Ecology and Evolution* spanned countries (shaded in grey) around the globe and species from multiple classes and phyla. [Correction added on 7 February 2025, after first online publication: An arrow in Graphical Abstract/Figure 1 has been repositioned]

## 1 Species' Behaviour

Camera traps provide unique insights into species' behaviour, as they allow observations without the potential intrusive effects of an in‐person observer (Caravaggi et al. 2017, 2020). Research on aspects of behaviour, such as parental investment in offspring care, can shed new light. In the Antarctic, Adélie penguins ( *Pygoscelis adeliae* ) reproduction varies with environmental factors, but also the degree of maternal investment in building nests, from limited resources (stones) (McLatchie et al. 2024). Camera traps revealed that occupying nests earlier, resulting in earlier clutch initiation, was associated with high‐quality nests which increased reproductive success. Larger nests were advantageous for successful breeding, and these nests were more likely to be created by penguins that built early (McLatchie et al. 2024). Environmental factors mediated this relationship, and this innovative study illustrates what can emerge from future research that combines behaviour with environmental heterogeneity.

Behaviour is also an important part of risk avoidance, with many studies showing humans impose a perceived risk to mammals, even via non‐consumptive activities such as recreation (Taylor and Knight 2003; Larson et al. 2016). In British Columbia, Canada, Fennell et al. (2023) examined spatiotemporal avoidance of humans by eight large mammals in an alpine protected area. They observed spatial co‐occurrence between ungulates and recreation consistent with the human shield hypothesis (Berger 2007), but not the expected consequent spatial segregation between larger carnivores and humans; instead, carnivores (and herbivores) temporally displaced from human recreationists (Fennell et al. 2023). Scaling up (sensu Steenweg et al. (2017)) from this landscape to 10 landscapes across the Canadian west, Granados et al. (2023) conducted a similar analysis. They used hierarchical models to quantify the influence of recreation and landscape development (roads and logging) on ungulate and carnivore site use. Across this vast heterogeneous space, they found limited consistent support for the human shield hypothesis, with positive and negative responses to recreationists and landscape disturbance across species (Granados et al. 2023). A signal of temporal segregation also ran contrary to predictions, as temporal overlap between people and deer increased with road density. Such macroecological studies—made possible by networking across camera‐trap arrays (Gallo et al. 2019; Fidino et al. 2021; Barnas et al. 2024)–are revealing much about large‐scale departures from phenomena observed at smaller scales.

Territorial defence is another behavioural mechanism by which risk is minimised, sometimes by passive means such as scent‐marking. Previously, most scent‐marking research focused on the transmitting individual, with the receivers harder to observe; camera traps have filled this gap. In northern Botswana, camera traps sampling latrines scent‐marked by African wild dogs ( *Lycaon pictus* ) were used to classify behaviours of sniffing (less aggressive response) or overmarking (aggressive response) (Claase et al. 2024). Wild dogs exhibited a “dear enemy” behavioural response to competitors, with less aggressive responses to neighbours than to strangers. This response changed with increasing size of the responding pack, switching to responding more strongly to neighbour scent marks (“nasty neighbour hypothesis”) (Claase et al. 2024).

Animals' temporal activity pattern is another component of behaviour illuminated by camera traps, which can sample continuously through the diel cycle (Frey et al. 2017). In Rhode Island, USA, Mayer et al. (2023) used a multi‐state diel occupancy‐modelling framework informed by camera trap data to investigate how 14 mammal species temporally responded to anthropogenic landscape development. All species' diel activity changed with respect to the magnitude of development and season, illustrating plasticity in this trait, a likely adaptation to perceived risk (Mayer et al. 2023). This body of research illustrates the complexity of species‐human interactions and their changing effects across contexts, a marked challenge for ecologists in the coming century.

## 2 Communities

One of the greatest strengths of camera traps is their ability to observe multiple syntopic species simultaneously (Rovero and Zimmermann 2016)–as opposed to GPS collars, which are typically restricted to one or two species. Thus, community composition can be observed like never before; this is now being done in regions previously rarely researched. In Nepal, Regmi et al. (2023) examined mammal community composition and observed that native species occurrence increased with forest cover but also livestock detections while declining with proximity to human settlements. They provided insights on 15 rarely studied species, including blue sheep ( *Pseudois nayaur* ) and giant flying squirrels ( *Petaurista magnificus* ) and their spatial relationships to human activity in this diverse but understudied region (Regmi et al. 2023).

In South Africa, carnivore communities live in uneasy coexistence, competing and subject to intraguild mortality. Camera traps placed at elephant ( *Loxodonta Africana* ) carcasses revealed consistent association and shared peak activity periods between black‐backed jackals (*Lupulella mesomelas*) and spotted hyaenas ( *Crocuta crocuta* ), indicating potential resource sharing (Honiball et al. 2024). Conversely, analysis showed spatial and temporal segregation between these species and lions ( *Panthera leo* ), suggesting the latter dominates scavenging opportunities. Parsing apart dominance and intraguild dynamics is a key area of investigation facilitated by camera traps (Honiball et al. 2024).

In Serengeti National Park, Tanzania, van den Bosch et al. (2023) examined aardwolves ( *Proteles cristata* ) and aardvarks ( *Orycteropus afer* )—both nocturnal insectivores—to examine spatiotemporal co‐occurrence of these two potential competitors. In fact, multispecies occupancy modelling showed a high degree of spatial and temporal overlap in the two species, with evidence that the two are commensals: aardvarks increase food accessibility for aardwolves (van den Bosch et al. 2023). In Mozambique's Gorongosa National Park, Grabowski, Phillips, and Gaynor (2024) used camera traps to explore patterns of spatial and temporal niche partitioning among mesocarnivores: large‐spotted genet ( *Genetta maculata* ), African civet ( *Civettictis civetta* ), honey badger ( *Mellivora capensis* ), and marsh mongoose ( *Atilax paludinosus* ). Statistical analyses found no evidence of spatial or temporal partitioning and, in fact, indicated co‐occurrence between civets and mongooses, suggesting limited competition and syntopy facilitated by different diets (Grabowski, Phillips, and Gaynor 2024). Differing outcomes across taxa and ecosystems among these studies reveal the complexity of the ways in which spatiotemporal strategies affect community composition and species' coexistence.

Many modern biological communities contain invasive species, a major threat to native biodiversity (Rosenzweig 2001). In Tianjin, China, Li et al. (2023) used camera traps and generalised additive mixed models to see how invasive species ( *Canis lupus familiaris* and *Felis silvestris* ) and three indigenous mammals (Siberian weasels, *Mustela sibirica* ; Amur hedgehogs *Erinaceus amurensis* , and Tolai hares, *Lepus tolai* ) densities changed along a gradient of urbanisation. Densities of cats, weasels, and hedgehogs increased with urbanisation, and that green spaces in urban areas were important predictors of density (Li et al. 2023)–a critical finding in a time when urban areas are growing rapidly (Seto et al. 2011; Simkin et al. 2022). This urbanisation effect was also examined in Australia by Alting et al. (2024) for dingoes ( *Canis dingo* ). Camera trapping across a gradient of urban development sought evidence for the resource dispersion hypothesis, which suggests concentrated resources should increase density and shrink home‐range sizes. Both were corroborated for dingoes, illustrating the effects of urban resource subsidies on this species (Alting et al. 2024), which has such marked repercussions for Australian native and non‐native community diversity (Johnson, Isaac, and Fisher 2007).

## 3 Predator–Prey Ecology

Prior to camera traps, predator–prey ecology was restricted largely to radiocollar studies in which the predation rates of predator or prey (or rarely both) were observed. The spatiotemporal outcomes of predator–prey interactions have been harder to discern until the multispecies sampling capability of camera traps bridged that gap. The fixed spatial design of camera traps has allowed questions about how–for example–anthropogenic resource extraction affects predators and prey in space and time. Boczulak et al. (2023) deployed camera traps across western Alberta, Canada and found that wolves ( *Canis lupus* ) avoid anthropogenic landscape features created by resource extraction, presumably due to perceived risk—unless large ungulate prey occur at those patches, at which point wolves switch to strong selection for these features. The interaction between the novel features and prey availability is critical for understanding how wolves respond to landscape change (Boczulak et al. 2023). Conversely, cougars ( *Puma concolor* ) in eastern Alberta, Canada showed no response to anthropogenic resource development. Cougars were positively associated only with native prey—particularly snowshoe hares ( *Lepus americanus* ) (Gaston et al. 2024). Invasive white‐tailed deer ( *Odocoileus virginianus* ) and anthropogenic features played no discernible role, defying predictions (Gaston et al. 2024), illustrating how difficult it is to extrapolate ecological conclusions among species, even within the same guild.

Scavenging, as a special case of predator–prey interactions, is a prime subject for camera‐trap research, which can also yield insights on competition. In northeastern Norway, Lacombe et al. (2024) sought evidence of asymmetric competition outcomes between the Arctic fox ( *Vulpes lagopus* ) and the red fox ( *Vulpes vulpes* ) at camera sites supplied with carrion. Both species tended to avoid sites occupied by the heterospecific, suggesting competition avoidance, but without either species clearly benefitting. Geographic variation and an effect of rodent (prey) abundance also affected occupancy dynamics, yielding insights into the delicate interplay between potentially competing species in cold northern systems (Lacombe et al. 2024).

## 4 Species at Risk

There is inherent risk in invasive research such as live trapping, even more so to threatened or endangered species. Mortality is always a concern, and moreover, catchability often declines with rarity. Camera traps provide a non‐invasive sampler for these rare species. In the United Kingdom (UK), Shannon, Valle, and Shuttleworth (2023) used camera traps to monitor endangered red squirrel ( *Sciurus vulgaris* ) populations, showing that image‐capture rate correlated well with live‐capture rate. They used camera traps to identify forest characteristics associated with greater red squirrel densities to inform conservation planning, particularly crucial in the UK's much deforested landscapes (Shannon, Valle, and Shuttleworth 2023).

## 5 Climate Change Adaptation

Climate change is expected to generate wholesale changes in species distributions (Pereira, Navarro, and Martins 2012), and much research has been devoted to species' ability to adapt to change (Boutin and Lane 2014). Among northern mammals, pelage change is a primary adaptation to variably snowy environments, and questions arise whether phenological mismatches between pelage and snow may occur. In Norway, Stokes et al. (2023) used a large network of camera traps to observe mountain hare ( *Lepus timidus* ) coat colour along climatic gradients. Mountain hares at higher latitudes and altitudes retained winter white coats longer than did hares at lower latitudes and altitudes. Moreover, hares in coastal climates retained white pelage longer than did inland hares, suggesting adaptation to variable climatic conditions (Stokes et al. 2023).

## 6 Aquatic and Marine Systems

Camera trap research spans aquatic and marine systems as well, with continuous video feed being the more commonly used “trap” (Willis, Millar, and Babcock 2000; Whitmarsh, Fairweather, and Huveneers 2017; Bulger, Volpe, and Fisher 2019). In a Red Sea coral reef, Lilkendey et al. (2024) examined feeding mechanics and foraging energy expenditure of herbivorous brown surgeonfish ( *Acanthurus nigrofuscus* ) and yellowtail tang ( *Zebrasoma xanthurum* ). Here, “camera trapping” included remote underwater stereo video processed with AI‐driven recognition, classification, and 3D tracking. Both species exerted marked grazing pressure on the reef in spite of low biomass. Brown surgeonfish exhibited a specialist strategy, and yellowtail tangs a generalist strategy, suggesting niche differentiation while maintaining similar energy efficiency. The combination of marine video with assisting technologies showcases the revolutionary potential of remote biotic sensing in marine environments (Lilkendey et al. 2024).

Exciting opportunities also lay in the land‐water interface. In the eastern USA, Sullivan, Rittenhouse, and Vokoun (2023) used camera traps to sample cold‐water patches in riverine systems. These refuges are important aggregators of fish seeking thermal refuges, but there they become susceptible to mortality. Avian and mammalian predators were pervasive at riverine thermal refuges, suggesting these patches concentrate opportunities for predator–prey interactions (Sullivan, Rittenhouse, and Vokoun 2023). In southern Mexico, Delgado‐Martínez et al. (2023) examined the use of ephemeral water bodies by birds and mammals in seasonally dry tropical forests. Camera traps deployed on surface pools and tree holes recorded species diversity and also behaviour. Terrestrial and larger mammalian species preferentially used surface water bodies, whereas arboreal and scansorial small and medium mammals were more common in arboreal water bodies; the two complementary sources thus facilitate gamma diversity (Delgado‐Martínez et al. 2023).

In Finland, Holopainen et al. (2024) deployed camera traps on artificial nests (with supplied eggs) around wetlands to examine predation pressure on ground‐nesting boreal ducks. Predation risk was higher in nests surrounded by agricultural land than forested land. Shoreline nests had higher predation than artificial nests further away from water bodies; higher predation pressure in observed agricultural landscapes may contribute to duck population declines in Finland (Holopainen et al. 2024). In summary, this growing body of work illuminates the importance of research spanning the land‐water interface, rarely studied ecosystems ripe for future discoveries.

## 7 Plants and Insects

Homeothermic mammals were a primary focus of camera traps, but the taxonomic breadth of camera‐based inquiry has expanded greatly with the use of video and frequent timelapse photos. Research on plant phenology (Hofmeester et al. 2020; Sun et al. 2021) and plant‐insect interactions (Naqvi et al. 2022) is very new but holds exciting potential. In California, USA, Simokat et al. (2024) examined the pollinator community of the rare endemic Encinitas baccharis ( *Baccharis vanessae* ). They compared video sampling with in‐person focal observations to compare the efficacy of the two methods. They discovered that Encinitas is attended by a diversity of insect groups. Focal observations underreported insect activity by approximately half compared to camera traps, but camera trap images were too low‐resolution to discern visually similar groups. Cameras had the benefit of recording nocturnal insect activity; however, it was dominated by Lepidopterans (Simokat et al. 2024).

In another unique study, Gao et al. (2024) used ground‐facing camera traps and timelapse imagery to quantify soil‐dwelling invertebrate diversity in eastern China. Camera traps were successful in quantifying Formicidae, Diplopoda, Gastropoda, Araneae, Coleoptera, Orthoptera, Chilopoda, Gastropoda, and Oligochaeta. Like many scale problems in ecology (Levin 1992), invertebrate abundance, richness, and density were all quite sensitive to frame size (shooting area), and the authors offer an effective protocol for future research (Gao et al. 2024).

As image resolution increases and memory gets smaller and cheaper, the use of camera traps for plant‐insect studies is sure to accelerate in the near future.

## 8 The Broad Frontier of Camera‐Trap Research

The field of ecology is a nascent human endeavour, having newly evolved from its natural philosophy and biogeography parentage only in the past century. It is still developing a coherent theoretical framework, and it suffers greatly from problems of scale, middle‐number problems, lack of unified theories, and general laws (Peters 1991; Levin 1992; Lawton 1999; Allen and Starr 2019). Nonetheless, ecologists march on, daily revealing new insights into species and systems that inexorably accumulate. Camera trap research plays a new role in this endeavour, with discoveries coming fast around the world. With networks of camera‐trap researchers as well as citizen scientists pooling data, research is scaling up from local to continental scope (Steenweg et al. 2017) in efforts such as Snapshot USA (Kays et al. 2022), Canada's WildCAM (Granados et al. 2023), and Snapshot Safari (Pardo et al. 2021). The future will reveal ecological insights from local to global scales to reveal macroecological patterns and laws to help us frame our concepts of natural system function. Ecology and Evolution will promote these endeavours by continuing to publish and highlight camera‐trapping papers, adding to this virtual issue online.

## Author Contributions

**Jason T. Fisher:** conceptualization (lead), investigation (lead), project administration (lead), resources (lead), software (lead), visualization (supporting), writing – original draft (lead), writing – review and editing (lead).

## Conflicts of Interest

The author declares no conflicts of interest.

## Data Availability

The author has nothing to report.
